# Oral consumption of α-linolenic acid increases serum BDNF levels in healthy adult humans

**DOI:** 10.1186/s12937-015-0012-5

**Published:** 2015-02-26

**Authors:** Mahmoudreza Hadjighassem, Behnam Kamalidehghan, Nima Shekarriz, Argavan Baseerat, Nima Molavi, Masoud Mehrpour, Mohammad Taghi Joghataei, Mahdi Tondar, Fatemeh Ahmadipour, Goh Yong Meng

**Affiliations:** 1 Cellular and Molecular Research Center, Iran University of Medical Sciences, Tehran, Iran; 2 Department of Pharmacy, Faculty of Medicine, University of Malaya (UM), Kuala Lumpur, Malaysia; 3 Faculty of Medicine, Iran University of Medical Sciences, Tehran, Iran; 4 Department of Neurology, Faculty of Medicine, Iran University of Medical Sciences, Tehran, Iran; 5 Brain and Spinal Cord Research Center, School of Advanced Medical Technologies, Tehran University of Medical Sciences, Tehran, Iran; 6 Department of Anatomy, Faculty of Medicine, Iran University of Medical Sciences, Tehran, Iran; 7 Department of Biochemistry and Molecular & Cellular Biology, Georgetown University, Washington, USA; 8 Department of Animal Science, Faculty of Veterinary Medicine, Universiti Putra Malaysia, Serdang, Malaysia

**Keywords:** α-Linolenic acid, Oral consumption, BDNF level, Neuroprotective effects, Stroke

## Abstract

**Background aims:**

Dietary omega-6 and omega-3 fatty acids have remarkable impacts on the levels of DHA in the brain and retina. Low levels of DHA in plasma and blood hamper visual and neural development in children and cause dementia and cognitive decline in adults. The level of brain-derived neurotrophic factors (BDNF) changes with dietary omega-3 fatty acid intake. BDNF is known for its effects on promoting neurogenesis and neuronal survival.

**Methods:**

In this study, we examined the effect of the oral consumption of α-Linolenic acid (ALA) on blood levels of BDNF and Malondialdehyde (MDA) in healthy adult humans. 30 healthy volunteers, 15 men and 15 women, were selected randomly. Each individual served as his or her own control. Before consuming the Flaxseed oil capsules, 5cc blood from each individual was sampled in order to measure the plasma levels of BDNF and MDA as baseline controls. During the experiment, each individual was given 3 oral capsules of flaxseed oil, containing 500mg of alpha linolenic acid, daily for one week. Then, plasma levels of BDNF and MDA were tested.

**Results:**

The plasma levels of BDNF and MDA significantly (P < 0.05) increased in individuals who received the oral capsules of ALA. Plasma levels of BDNF increased more in the women in comparison with the men.

**Conclusion:**

ALA treatment could be a feasible approach to reduce size of infarcts in stroke patients. Thus, ALA could be used in adjunction with routine stroke therapies to minimize brain lesions caused by stroke.

## Introduction

The long chain omega-3 fatty acid with 6 double bonds and 22 carbons, docosahexaenoic acid (DHA), is the most abundant omega-3 fatty acid in the mammalian central nervous system. DHA is concentrated in the visual units of retina and membrane lipids of the brain grey matter. Levels of DHA increase during mammalian development and reduce by aging [1-5].

Many researchers have shown that dietary ω-6 and omega ω-3 fatty acids have remarkable impact on the levels of DHA in brain and retina [6,7]. Furthermore, according to many epidemiological studies, low levels of DHA in plasma and blood hamper visual and neural development in children and cause dementia and cognitive decline in adults [8-22].

A growing body of evidence indicates that omega-3 fatty acids have neuroprotective impact on the nervous system. These fatty acids influence the levels of neurotrophins, molecules that increase neuronal growth and survival. Among neurotrophins, the level of brain-derived neurotrophic factor (BDNF) changes with dietary omega-3 fatty acids intake [23,24]. BDNF is known for its effects on promoting neurogenesis and neuronal survival [25,26].

The α-Linolenic acid (ALA; 18:3n - 3) is a polyunsaturated omega-3 fatty acid that has several neuroprotective effects [27-32]. In this study, we measured the plasma levels of BDNF and MDA in two groups of healthy participants, those who received ALA and those who did not.

## Material and methods

This study was evaluated and approved by the Ethical Committee of the Tehran University of Medical Sciences. Thirty healthy volunteers, fifteen men and fifteen women, were selected randomly. They read and signed a consent form prior to enrolment in this study. These individuals had Body Mass Indexes (BMI) of less than thirty, similar low-fat diets, and no underlying diseases such as diabetes or high blood pressure. Because effective doses of ALA for increasing BDNF levels are unknown, each individual served as his or her own control.

Before consuming the Flaxseed oil [(Swiss, Canada) (Table 1)], 5cc blood from each individual was sampled in order to measure the plasma levels of BDNF and MDA as baseline controls. During the experiment, each individual was given 3 oral capsules of flaxseed oil, containing 500mg of ALA, daily for one week. Then, Plasma levels of BDNF and MDA were assessed using BDNF Emax*®* ImmunoAssay (Promega) and colorimetric Assay (Oxford Biomedical Research) kits according to the manufacturer’s protocols, respectively.

**Table 1:** **Flax seed oil capsules**

Flax seed oil	ALA	Company
1000 mg	530 mg	Swissherbal

Each 1000 mg flax seed oil capsule contained 530 mg ALA.

### Statistical analysis

GraphPad prism5 was applied to compare the levels of BDNF and MDA, after taking the capsules for one week, to their baselines. Numerical data are presented below as means ± SEM. Statistical testing used Paired t-test analysis. Each test was performed at least two times and P < 0.05 was considered significant.

## Results

**Figure 1:**
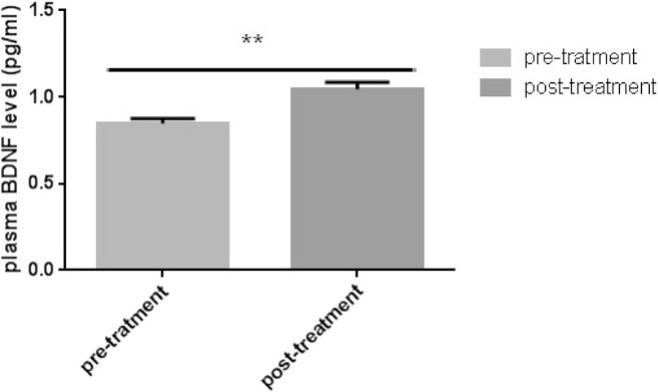
**Plasma level of BDNF.** Plasma Levels of BDNF were detected by ELISA. Data were analyzed by GraphPad prism5. Bars refer to the Mean and SEM with P < 0.05.

**Figure 2:**
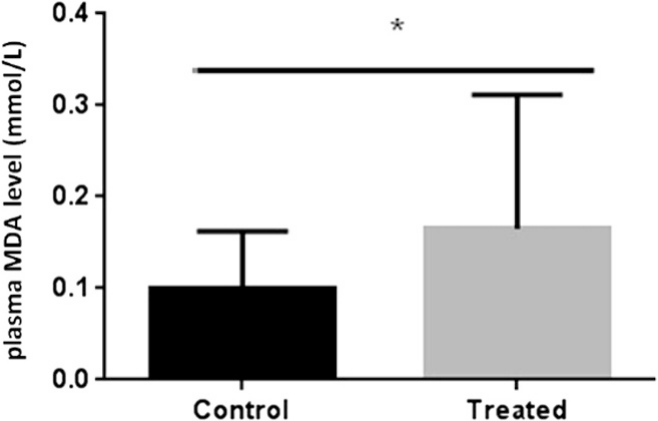
**Plasma level of MDA.** Plasma Levels of MDA were measured before (cont) and after intake of Flax seed oil. Bars represent the SEM with P < 0.05.

This study revealed that plasma BDNF levels significantly (P < 0.05) increased in individuals who received the oral capsules of ALA (Figure 1). In order to determine whether or not this phenomenon was associated with peroxidation of fatty acids, plasma levels of MDA in the ALA group were measured, and they notably (p < 0.05) increased. (Figure 2)

.

**Figure 3:**
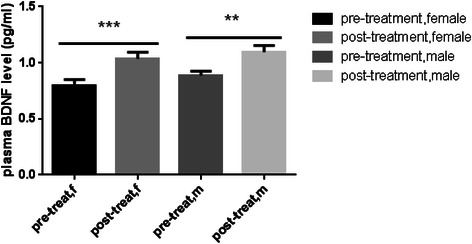
**Sex dependency of BDNF change.** BDNF levels were compared between the males and females before and after receiving the capsules.

In addition, plasma levels of BDNF increased more in the women in comparison with the men. Although we observed a significant positive trend in increasing the BDNF levels in the men (P = 0.01) (Table 2, Figure 3).

**Table 2:** **Data analysis of serum BDNF levels in males and females**

Sex			Pretreatment	Post-treatment	P value
	Mean	Female	0.7987	1.036	0.005
	Std.Deviation		0.1740	0.2035	
	Mean	Male	0.8872	1.096	0.01
	Std.Deviation		0.1270	0.1986

## Discussion

The neuroprotective roles of ALA have been reported in several stroke studies [27,31-36]. Recently, several molecular and clinical studies emphasized on the therapeutic potential of Omega-3 polyunsaturated fatty acids for treating a number of neurological and psychiatric diseases. Nevertheless, the mechanisms underlying these effects are still poorly understood.

In 2009, Blondeau et al. showed that subchronic ALA injections in mice induced neurogenesis in the hippocampus, increased *in vivo* and *in vitro* BDNF expression, promoted Neural Stem cell (NSCs) proliferation and synaptogenesis, enhanced synaptic vesicle fusion and protein levels, and induced antidepressant-like behavior. Furthermore, they observed that pre- and post-treatments with repeated ALA injections decreased the infarc volumes and mortality caused by middle cerebral artery occlusion (MCAO) [37]. Nguemeni et al. in 2010 reported that dietary supplements of ALA in an enriched rapeseed oil diet could significantly reduce the MCAO-induced mortality rate and infarct volumes in mice [38]. In light of these studies on ALA, we examined the effect of dietary consumption of ALA on the blood levels of BDNF and MDA. To the best of our knowledge, this is the first study on healthy adult humans that measured both BDNF and MDA levels, used oral consumption of ALA, and determined sex differences in response to ALA intake. The results demonstrated that the levels of BDNF and MDA both increased in individuals who took ALA.

Neurotrophins are small proteins that are crucial for neuronal differentiation, growth, survival, and plasticity [39]. Nerve growth factor (NGF), brain-derived neurotrophic factor (BDNF), neurotrophin-3 (NT-3), and neurotrophin-4/5 (NT-4/5) are members of the mammalian neurotrophin family. The impact of these molecules on the nervous system is mediated by the tropomyosin receptor kinase (Trk) receptors and membrane-bound receptor tyrosine kinases that activate a number of cell signaling pathways which are linked to growth, differentiation, and survival [40]. The importance of neurotrophin signaling in brain development is well elucidated with findings that showed that knockout mice for any of the neurotrophins or their receptors were fatal or exhibited severe neural defects [41]. Neurotrophin signallings have important roles in the survival and integration of new neurons. For instance, BDNF triggers the TrkB receptor tyrosine kinases. BDNF also increases the number and survival of NSCs in the subventricular zone (SVZ) and olfactory bulbs [42,43]. Likewise, knocking down the TrkB receptors or disrupting the BDNF signaling pathway in dentate gyrus progenitors can lead to the formation of shorter dendrites, reduced spine, and eventually death [44].

BDNF signaling promotes the survival of newly-generated neurons. In addition, defects in this pathway are associated with decreased neuronal survival and neurogenesis as well as the incidence and progression of several neurological disorders, such as schizophrenia, bipolar disorder, Alzheimer’s disease, and age-related cognitive decline [43,45,46]. Furthermore, BDNF indirectly increases the transcription of *Bcl-w* gene, an anti-apoptotic member of the Bcl-2 family [47]. Thus, BDNF decreases neuronal apoptosis. In addition, BDNF increases adhesion, migration, and survival of neurons. This neurotrophic molecule also enhances neurogenesis, synaptic plasticity, and neuronal differentiation through the BDNF/TrkB-TK+ signaling pathway, an important pathway for neuronal viability and function [48-57].

ALA treatment can be beneficial for the treatment of many neurological diseases, particularly stroke, which is the third leading cause of death worldwide [58,59]. Our findings were in accordance with the previous studies, confirming that ALA increases the expression of BDNF. Considering the neuroprotective and neurotrophic characteristics of BDNF, ALA treatment could be a feasible approach to reduce infarct size in stroke patients. Thus, ALA could be used in adjunction with routine stroke treatments to minimize lesions caused by stroke. Further research could attempt to replicate the present findings with a larger sample size. Furthermore, studying the molecular mechanisms underlying the positive effects of ALA on the nervous system might also be helpful. Future research can investigate the effects of ALA intake on stroke patients.

## Abbreviations

ALA: α-Linolenic Acid
BDNF: Braine-derived Neurotrophic Factor
DHA: Docosahexaenoic acid
MDA: Malondialdehyde
